# Shift work and risk of skin cancer: A systematic review and meta-analysis

**DOI:** 10.1038/s41598-020-59035-x

**Published:** 2020-02-06

**Authors:** Einas Yousef, Noha Mitwally, Noha Noufal, Muhammad Ramzan Tahir

**Affiliations:** 1 Department of Basic Medical Sciences, College of Medicine, Dar Al Uloom University, Riyadh, KSA Saudi Arabia; 2 Histology Department, Faculty of Medicine, Menoufia University, Shebin Elkom, Egypt; 3 Pathology Department, Faculty of Medicine, Suez Canal University, Ismailia, Egypt; 4 Apotex Inc., Toronto, Canada

**Keywords:** Skin cancer, Risk factors

## Abstract

Shift work with circadian disruption has been considered as a carcinogenic risk factor for skin cancer. The few prior studies that investigated the association between shift work and skin cancer have inconclusive results. Our main objective was to evaluate the associations between shift work and the risks of different types of skin cancer. We systematically searched PubMed, Web of Science, Cochrane Library, EMBASE and Science Direct until October 2018 for studies that included a relationship between shift work and skin cancer. Our search yielded 193 articles and 9 studies met the criteria for our review. The included studies involved 3,579,147 participants and 17,308 skin cancer cases. Overall, ever shift work, was associated with increased risk of melanoma (RR = 1.10, 95% CI = 1.05–1.16) and a significant decrease in the risk of BCC (RR = 0.90, 95% CI = 0.88–0.93). No association between shift work and the risk of SCC was detected. Interestingly, our dose response analysis demonstrated that the risk of melanoma cumulatively increases by 2% for every year of shift work (RR = 1.02; 95% CI = 1.00–1.03). In conclusion, shift work is associated with increased risk of melanoma and deceased risk of BCC. Further studies are needed to confirm our findings and to elucidate the related potential biological mechanisms.

## Introduction

Shift work is increasingly common, especially in industrially developed countries. It includes a wide range of schedules, such as evening work; night work; split, extended or rotating shifts; weekend work; and on-call work^1,2^. According to the most recent data from the Bureau of Labour Statistics, approximately 15 million Americans work night shifts, and that number is expected to grow rapidly^3^. Although such work imposes significant costs on workers, including many hazardous impacts not only on their relationships, social lives and sleep patterns but also on their overall health, it is unavoidable and is taken for granted by many companies. Indeed, the International Agency for Research in Cancer (IARC) considered shift work that involves circadian disruption to be a probable carcinogenic factor in humans (Group 2 A carcinogen)^4^. The desynchronization that occurs in circadian rhythms with respect to sleep cycles and melatonin production predisposes employees to have a higher susceptibility to cardiovascular, neuropsychiatric and endocrine disorders and to the development of neoplastic growths^5^. A recent meta-analysis showed that shift work increases the risks of multiple primary cancers, such as breast, prostate and digestive system cancers^6^.

According to the last annual report by the Skin Cancer Foundation, skin cancer is the most commonly diagnosed cancer in the USA^7^. It can be classified into melanoma skin cancer, basal cell carcinoma (BCC) and squamous cell carcinoma (SCC). The last two subtypes are grouped together as non-melanoma skin cancer which is the most frequently diagnosed cancer in white populations^8^. Both BCC and SCC have a good prognosis when detected in their early stages. While most non-melanoma skin cancer are rarely fatal, they can result in considerable morbidity which presents an increasing burden on the healthcare services^9^. On the other hand, while invasive melanoma accounts for only a small percentage (~4%) of all skin cancer cases, it is responsible for the vast majority of deaths due to skin cancer^8^. The main risk factor for all types of skin cancer is exposure to ultraviolet light. However, many other risk factors have been reported to predispose patients to skin cancer, such as family history of the disease, fair skin and shift work^10,11^.

Previous studies that examined the relationship between shift work and skin cancer risk have provided inconclusive results. Some studies have demonstrated a significant association between shift work and skin cancer^12–15^, while others have failed to reveal any significant correlation^16,17^. In this context, identification of the associations between shift work and the risks of different types of skin cancer would be of the utmost interest. In the present study, we conducted a meta-analysis to summarize the results of published case-control and cohort studies exploring the association between shift work and the risk of skin cancer. More specifically, we sought to assess the effect of increased duration of shift work on the risk of skin cancer.

## Results

### Literature search and description of the selected studies

**Figure 1:**
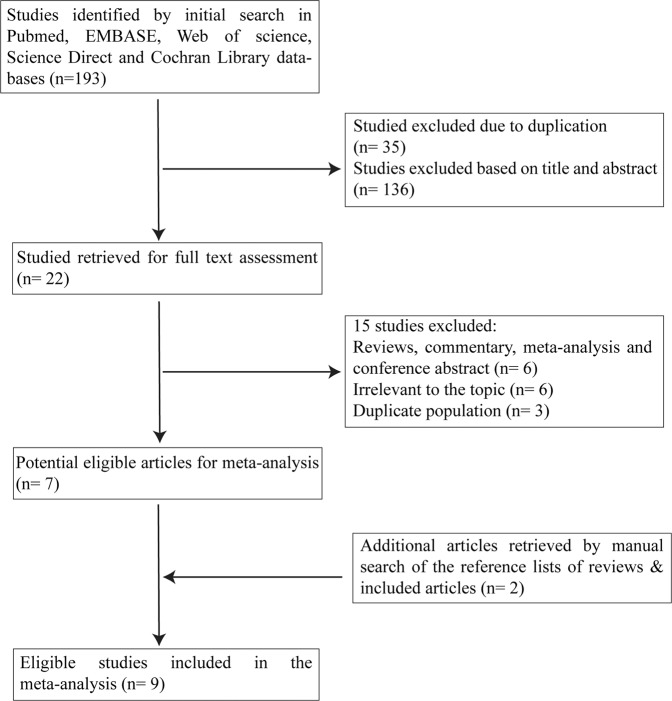
Flow chart for the process of eligible articles selection.

The detailed process of eligible article identification and selection is depicted in Fig. 1. Our initial literature search identified 193 articles; 171 articles were excluded either because of duplication (n = 35) or because they were found to be not related to the topic after checking the titles and abstracts (n = 136). Twenty-two studies were retrieved for further full-text assessment. Of the remaining twenty-two records, fifteen articles were excluded for the following reasons: six articles were reviews, commentaries, meta-analyses and conference abstracts; six were irrelevant to our topic; and three reported duplicate populations. Two articles were retrieved after a manual search of the reference lists of reviews and included studies and were incorporated in this meta-analysis. Finally, our study included a total of nine articles^13–21^ evaluating the associations between shift work and the risks of different types of skin cancer that were published before 30 October 2018 with a total of 17,308 skin cancer cases and 3,579,147 participants.

The descriptive data from the studies included in our meta-analysis are summarized in Table 1. Among the nine included studies, four were conducted in the USA, three in Nordic countries, one in Canada and one in Germany. According to the study design, eight were cohort studies, and one was a case-control study. Five studies included a female population, two included a male population and two included both males and females. However, in the Cohen 2015 study, we included only the male population, as the female cohort from the Nurses’ Health Study II was already used in a more recent study that included a larger number of participants and controlled for many more variables^15,18^. Approximately half of the included studies were conducted among nurses and health professionals, one study was conducted among flight attendants, and three studies were conducted among different types of workers. In terms of skin cancer types, nine studies reported melanoma, two studies reported SCC and two studies reported BCC. Of note, articles that included different types of skin cancers, sexes and multivariate models were considered independent entries. The number of adjustment factors in the individual studies ranged from 2 to 22. Eight studies adjusted for >3 covariables and only one adjusted for ≤3 covariables. For exposure assessment, four studies used databases, three adopted questionnaires, one study used interviews, and one study used all three instruments.

**Table 1:** Main characteristics of included studies on the association of shift work and the risks of different types of skin cancers.

Study	Study design	No. of cases/No. of subjects	Sex	Age (years)	Occupation	Type of skin cancer	Range of shift work	Definition of shift work	Exposure assessment	Variables of adjustments	NOS
Heckman, 2017, USA	Cohort	4854/74323	Female	25–42	Nurses	BCC, SCC, Melanoma	Never vs ever	1989 questionnaire: at least 3 nights/month + days or evenings in that month. 1991, 1993, 1997 questionnaires: total number of months with rotating night shifts in the past two years. 2001 questionnaire: working permanent night shifts for ≥ 6 months. 2003–2005 questionnaire: total number of years having worked various types of shifts.	Questionnaire	Years of shift work, hours of sleep per night, sleep adequacy, sleepy days per week, snoring, restless legs syndrome, family history of melanoma, hours spent in sun per week ages 25–35, number of severe sunburns from ages 15 to 20, sunburn severity after ≥2 hours in the sun during childhood, artificial tanning frequency per year ages 25–35, annual UV at residence, moles on lower legs, natural hair color in adolescence, marital status, financial status on 10-rung ladder, body mass index Kg/m2, physical activity, smoking status, menopause status, postmenopausal hormones, oral contraceptive use, g/day alcohol intake and alternate healthy eating index.	8
Schernhammer, 2011, USA	Cohort	10 799/68 336	Female	–	Nurses	BCC, SCC, Melanoma	Never vs. 10+ years night shift work	At least 3 nights/month + days or evenings in that month.	Questionnaire	Multivariable model 1: age in 1 year increments, hair color at 20 years of age, family history of skin cancer, child and adolescence tendency to burns, number of palpable moles on arms and legs, and lifetime severe sunburns that blistered Multivariable model 2: additional adjustment for residence ultraviolet exposure level at birth, and at 15 and 30 years of age, average hours of sun exposure per week at 25–35, 36–59, and ≥60 years of age.	8
Parent, 2012, Canada	Case-control	94/799	Male	Mean = 52.9	Men who ever worked in any job involving night shift.	Melanoma	Never vs. ever	Working between 1:00AM and 2:00 AM for at least 6 months.	Interview	Age, ancestry, educational level, family income, and respondent status were included in all models. β-carotene, sports and/or outdoor activities	9
Kjaer, 2009, Denmark	Cohort	395/92140	Female	Mean = 27.1	Nurses	Melanoma	Never vs. 30+ years employment as a nurse	They consider working in a hospital usually involves shift work.	Database	Age at birth of first child in four categories, number of children in five categories, place of birth, and marital status in four categories	8
Yong, 2014, Germany	Cohort	27/12609	Male	Median = 39.5	Chemical workers	Melanoma	Never vs. ever	Two forms of shift work: In the 3×12 system with a sequence of shifts (day-night-off), a 12-hour day shift (06:00–18:00 hours) is followed 24 hours later by a 12-hour night shift (18:00–06:00 hours). After a day off, the employee returns to the day shift. The 4×12 schedule with a sequence of shifts (day-night-off-off) follows the same pattern except that there are two days off between the night shift and the next day shift.	Database	Age, smoking status, job level, employment duration	7
Pinkerton, 2018, USA	Cohort	125/4908	Female	Median = 48	Flight attendants	Melanoma	Never vs. 2000+ days of employment	Those who employed for at least one year as a flight attendant	Interview, questionnaire, Database	Age, year of birth, race/ethnicity, education and parity	7
Cohen, 2015, USA	Cohort	238/31929	Male & Female (male only included in our study)	Mean = 65.3–71.7	Health professionals	Melanoma	Short sleep duration vs. normal	They consider working in a hospital usually involves shift work	Questionnaire	Age, number of sun burns, moles, hair color, family history of melanoma, reaction to the sun, tanning, Caucasian ethnicity, UV flux, snoring.	6
Schwartzbaum, 2007, Sweden	Cohort	200/3250 787	Male & Female	≤19-≥60	Self-employed & employees in e.g. agriculture, forestry, industry & transport, service occupations & Military	Melanoma	Never vs. ever	Those who reported that their workplace had a rotating schedule with ≥3 possible shifts per day or had workhours during night (any hour between 01:00 and 04:00 AM) at least one day during the week preceding the interview.	Database	Age, socioeconomic status, occupational position in four categories, county of residence, marital status, and urbanization. Marital status and urbanization were not included in the final analyses, as they did not affect the results.	8
Lie, 2007, Norway	Cohort	576/43316	Female	>33-<80	Nurses	SCC, Melanoma	Never vs. ≥ 20 years of shift work	They consider working in a hospital usually involves shift work	Database	Age and calendar period	7

NOS: Newcastle-Ottawa Quality Assessment Scale; SCC: Squamous cell carcinoma; BCC: Basal cell carcinoma

### Quality evaluation

New castle Ottawa scale (NOS) was used to estimate the quality of the included studies according to a predefined set of criteria^22^. Overall, most studies were of good quality with a NOS score ranging from 6 to 9 with a mean of 7.6, as shown in Supplementary Table 1. Of all included studies, only one met all the criteria of NOS and scored as 9 and this was the case-control study^23^. Among the 8 cohort studies included in this meta-analysis, we found that the potential flaws were detected in three items of the population selection that includes “representativeness of exposed cohort”^14–18,20,21^, “selection of non-exposed cohort”^16,18^ and “outcome of interest history”^17,18,20,21^.

### Risk assessment and heterogeneity

**Figure 2:**
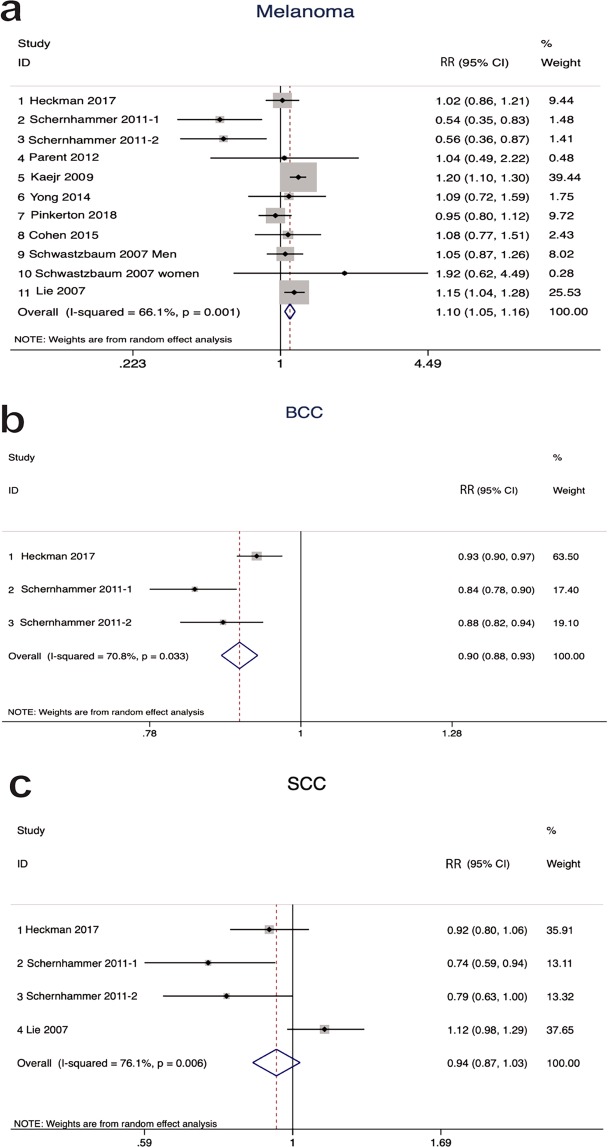
Forest plots depicting the risk estimates from included studies on the associations between shift work and risks of different types of skin cancer (**a**) Melanoma, (**b**) BCC, (**c**) SCC, RR: relative risk, CI: confidence interval. I^2^ is an indicator that used to determine the degree of heterogeneity in the meta-analysis. The horizontal lines and squares correspond to the 95% CI and to the study-specific RR. The area of the square represents the weight of each study. The dotted red-line and the diamond represents the 95% CI and the pooled RR.

We performed a meta-analysis and calculated the pooled effect estimates across the enrolled studies. The multivariate-adjusted relative risks (RRs) of each study were combined using the random effects model to identify the associations between shift work and the risks of different types of skin cancers (Fig. 2). The overall results indicated that shift work was potentially associated with a 10% increase in the risk of melanoma (RR = 1.10, 95% CI = 1.05–1.16) which was statistically significant. In contrast, a significant decrease (10%) in the risk of BCC (RR = 0.90, 95% CI = 0.88–0.93) and a non-significant decrease (6%) in the risk of SCC (RR = 0.94, 95% CI = 0.87–1.03) were detected. Notably, statistically significant heterogeneity among included studies was detected for melanoma (I^2^ = 66.1%, p < 0.001), BCC (I^2^ = 70.8%, p < 0.033), and SCC (I^2^ = 76.1%, p = 0.006).

### Subgroup and meta-regression analysis

To expand on the results obtained from the pooled RRs analysis and to explore the heterogeneity, subgroup analyses were performed for each type of skin cancer based on geographical regions (Americas, Europe), assessment of exposure (database, questionnaire, interview), quality score (low quality (NOS <7), high quality (NOS ≥ 7)), study design (cohort, case-control), occupation (nurses & health professionals, self-employed, workers, flight attendants), sex (male, female), and studies adjusted to the exposure to UV radiation in the multivariate analysis (yes, no).

For melanoma subtype, our results of the subgroup analysis stratified by geographical region suggest that shift work caused a significant increase in the risk of melanoma in Europe (RR = 1.16, 95% CI = 1.09–1.23), while no association was found in the America (RR = 0.84, 95% CI = 0.63–1.05). However, only the Americas group tended to have significant heterogeneity (p = 0.003, I^2^ = 74.73%). In the analysis stratified by exposure assessment, a positive relationship between the risk of melanoma and shift work was observed when conducting a study using a database (RR = 1.16, 95% CI = 1.09–1.23). Nevertheless, no relationship was found when conducting the study using questionnaires (RR = 0.79, 95% CI = 0.51–1.07) and in the interview group (RR = 0.95, 95% CI = 0.80–1.11). In the subgroup analyses stratified by quality score, study design, occupation, sex and using the exposure to UV radiation as a covariable in the multivariate analysis, non-significant associations were observed between shift work and an increased risk of melanoma in all subgroups, as shown in Table 2A.

**Table 2:** Stratified pooled risk ratio (RR) and 95% confidence interval (CIs) for the association between shift work and the risk of (A) melanoma, (B) BCC and (C) SCC.

*A. Melanoma*					
Subgroup	*Number of studies	RR (95% CI)	Heterogeneity		*P meta-regression*
			P	I^2^ (%)	
**Geographical region**					0.004
America	6	0.84 (0.63–1.05)	0.003	74.73	
Europe	5	1.16 (1.09–1.23)	0.630	0	
**Exposure assessment**					0.004
Database	5	1.16 (1.09–1.23)	0.630	0	
Questionnaire	4	0.79 (0.51–1.07)	0.001	80.46	
Interview	2	0.95 (0.80–1.11)	0.841	0	
**Quality score**					0.580
Low (NOS <7)	1	1.08 (0.71–1.45)	—	—	
High (NOS ≥ 7)	10	0.96 (0.80–1.13)	0.000	83.97	
**Study design**					0.879
Cohort	10	0.97 (0.81–1.13)	0.000	83.48	
Case-control	1	1.04 (0.17–1.91)	—	—	
**Occupation**					0.766
Nurses & Health professionals	6	0.94 (0.70–1.17)	0.000	91.15	
Self-employed	2	1.06 (0.86–1.25)	0.381	0	
Workers	2	1.08 (0.69–1.47)	0.919	0	
Flight attendants	1	0.95 (0.79–1.11)	—	—	
**Sex**					0.358
Male	4	1.06 (0.90–1.22)	0.998	0	
Female	7	0.93 (0.71–1.15)	0.000	90.40	
**Exposure to UV radiation					0.492
Yes	4	0.89 (0.61–1.18)	0.022	68.28	
No	7	1.01 (0.82–1.20)	0.000	84.76	
***B.BCC***					
Subgroup	* Number of studies	**RR (95% CI)**	**Heterogeneity**		***P**** meta-regression*
			**P**	**I**^2^ **(%)**	
**Exposure to UV radiation					0.065
Yes	2	0.91 (0.84–0.96)	0.158	49.73	
No	1	0.84 (0.78–0.90)	..	..	
***C.SCC***					
Subgroup	*Number of studies	**RR (95% CI)**	**Heterogeneity**		***P**** meta-regression*
			**P**	**I**^2^ **(%)**	
**Geographical region**					0.003
America	3	0.83 (0.71–0.95)	0.223	36.51	
Europe	1	1.12 (0.97–1.28)	..	..	
**Exposure assessment**					0.003
Database	1	1.20 (0.97–1.28)	..	..	
Questionnaire	3	0.83 (0.71–0.95)	0.223	36.51	
Interview	0	..			
**Exposure to UV radiation					0.765
Yes	2	0.87 (0.75–0.99)	0.259	21.49	
No	2	0.93 (0.56–1.31)	0.001	90.14	

NOS: Newcastle-Ottawa Quality Assessment Scale; RR: Risk ratio, CI: Confidence interval, UV: Ultraviolet.

*No. of studies: Some included studies have more than one model of multivariate analysis, others discussed different types of skin cancer and the risk in different gender separately, when calculating the number of studies; we considered each condition as a separate study.

**This subgroups analysis depends on using the exposure to UV radiation as a covariable during multivariable adjustment.

For BCC, in the subgroup analysis stratified by including UV radiation or not as a covariable in the multivariate analysis, A significant decrease in the risks of BCC with exposure to shift work was detected in both groups (Adjusted: RR = 0.91, 95% CI = 0.84–0.96) and (Unadjusted: RR = 0.84, 95% CI = 0.78–0.90) (Table 2B). For SCC, in the subgroup analysis stratified by geographical region, our results suggest that shift work caused a significant increase in the risk of SCC in the America (RR = 0.83, 95% CI = 0.71–0.95), while no relationship was found in Europe (RR = 1.12, 95% CI = 0.97–1.28). In the analysis stratified by exposure assessment, a positive relationship between the risk of SCC and shift work was observed when conducting a study using questionnaires (RR = 0.83, 95% CI = 0.71–0.95) while no relationship was found when conducting the study using databases (RR = 1.20, 95% CI = 0.97–1.28). In addition, in the subgroup analyses stratified by adjustment to UV radiation, a significant decrease in the risk of SCC with exposure to shift work was observed (RR =  0.87, 95% CI = 0.75–0.99). Nevertheless, no relationship was found when UV radiation was not included as a covariable in the multivariate analysis (RR = 0.93, 95% CI = 0.56–1.31) (Table 2C). Moreover, we carried out a meta-regression analysis to explore the possible sources of heterogeneity among the studied subgroups. Our results showed that geographical region and exposure assessment were potential sources of heterogeneity for melanoma and SCC subtypes.

### Dose-response meta-analysis

Four of our studies provided sufficient information to be included in the dose-response meta-analysis (included at least three categories of shift work, available number of cases and total subjects and risk estimates for each shift work category)^13,15,19,21^. All the four studies included melanoma, two studied SCC and only one for BCC. Our results demonstrated that every extra year of shift work is causing a significant increase of 2% of melanoma (RR = 1.02, 95% CI = 1.00–1.03). Moreover, exposure to shift work does not have an impact on the risks of both BCC (RR = 0.99, 95% CI = 0.93–1.05) and SCC (RR = 0.99, 95% CI = 0.98–1.01) (Table 3).

**Table 3:** Aggregated dose-response data of four studies investigating the association between shift work and different types of skin cancers.

Type of cancer	Author, Year	Dose (year)	No. of cases	RR (95% CI) for each subgroup	Dose response meta-regression RR (95% CI)
Melanoma	Heckman, 2017^15^	0	67	1	1.02 (1.01–1.03)
		1	54	0.85 (0.59–1.22)	
		4	45	0.84 (0.57–1.23)	
		8	28	1.13 (0.72–1.77)	
		20	18	0.95 (0.55–1.61)	
	Parent, 2012^19^	0	82	1	
		3	7	1.16 (0.44–3.11)	
		7	5	2.77 (0.89–8.58)	
		20	0	1	
	Kjaer, 2009^13^	3	44	1.6 (1.1–2.1)	
		7	63	1.3 (0.96–1.6)	
		15	48	1 (0.8–1.4)	
		25	97	1.4 (1.1–1.7)	
		40	143	1.1 (0.9–1.3)	
	Lie, 2007^21^	10	65	1	
		25	124	1.68 (0.84–3.36)	
		40	114	2.64 (1.12–6.26)	
		45	72	3.21 (1.12–9.24)	
BCC	Heckman, 2017^15^	0	1333	1	0.99 (0.93–1.05)
		1	1179	0.93 (0.86–1.01)	
		4	1032	0.96 (0.88–1.04)	
		8	416	0.83 (0.75–0.93)	
		20	348	0.83 (0.74–0.94)	
SCC	Heckman 2017^15^	0	106	1	0.99 (0.98–1.01)
		1	93	0.94 (0.71–1.24)	
		4	74	0.86 (0.63–1.16)	
		8	34	0.85 (0.57–1.26)	
		20	27	0.81 (0.53–1.25)	
	Lie 2007^21^	20	30	1	
		40	48	1.02 (0.32–3.22)	
		45	102	1.33 (0.37–4.77)	

### Sensitivity analysis

To evaluate the robustness of our results, we performed a leave-one-out-sensitivity analysis in which the meta‐analysis was serially repeated after sequentially leaving out exactly one study at a time. Our results demonstrated that none of the included studies unduly influenced the pooled estimate, as it remained stable, which confirmed that our findings were not driven by a single study. However, we did find that the heterogeneity was significantly reduced if the Schernhammer 2011^14^ study was omitted in the melanoma group, as the heterogeneity changed from (I^2^  =  66.1%, p <0.001) to (I^2^  =  15%, *p*  =  0.30). The same occurred in the other two groups of BCC and SCC after excluding the Heckman 2017^15^ Lie 2007^21^ studies, respectively, as heterogeneity changed from (I^2^ = 70.8%, p <0.033) to (I^2^ = 0%, p = 0.37) for BCC and from (I^2^ = 76.1%, p = 0.006) to (I^2^ = 31%, p = 0.23) for SCC.

### Publication bias

Egger’s regression test and a funnel plot were used to assess the existence of publication bias among the included studies. For melanoma, BCC and SCC, the funnel plots had symmetrical distributions, and no evidence of publication bias was detected by Egger’s test (p = 0.707, p = 0.248, p = 0.475 respectively) and the effect estimate was (RR = 0.974, 95% CI = 0.82–1.13), (RR = 0.888, 95% CI = 0.83–0.94) and (RR = 0.898, 95% CI = 0.73–1.06) respectively. For BCC, two possible missing studies were imputed by the trim-and-fill method and the effect estimate was (RR = 0.930, 95% CI = 0.87–0.99). However, no imputed studies were detected for both melanoma and SCC.

## Discussion

This meta-analysis summarized the results of one case–control and eight cohort studies that investigated the associations between shift work and the risks of different types of skin cancer and involved 3,579,147 participants and 17,308 skin cancer cases. Our analysis demonstrated positive associations between shift work and the risk of melanoma (RR = 1.10, 95% CI = 1.05–1.16). In contrast, an inverse significant association in the risk of BCC (RR = 0.90, 95% CI = 0.88–0.93) and a non-significant inverse association in the risk of SCC (RR = 0.94, 95% CI = 0.87–1.03) were detected. Our findings are consistent with previous observations by others reporting a significant association between a longer duration of night shifts and a decreased risk of BCC^15^ and an increased risk of melanoma among flight attendants, especially pilots^23–25^. In contrast, our results are clearly at odds with the observations of other studies that showed a reduced risk of melanoma in cohorts of chemical workers and nurses^14,15,20^. However, other previous studies have reported that no relationship can be detected between shift work and the risk of melanoma^17,18^.

Our dose-response analysis demonstrated that every extra year of shift work is causing a significant increase of 2% of melanoma. Similar results were demonstrated in several studies that showed higher risks of colorectal, breast, lung and prostate cancers with an increased duration of shift work^26–32^. The results presented herein, together with the findings of the previously mentioned studies, suggest that longer exposure to shift work increases the risks of different types of cancers. Moreover, one might assume that shift work is not a real problem in short-term, but its effect accumulates with time. One possible justification is that long-term circadian disruption associated with prolonged duration of shift work may play a role in development of melanoma.

The relationship between shift work and the risk of all types of skin cancer has some biological plausibility. One of the possible explanations is that shift workers are mostly exposed to light at night (LAN) that could trigger the inhibition of melatonin secretion with serious perturbations of the circadian rhythm^33^. Melatonin, which is considered a marker of the circadian rhythm, is a natural antioxidant with immunoenhancing properties^8,34^. Several *in vitro* studies speculated that melatonin derivatives exhibited antitumourigenic effects through their ability to inhibit melanoma and breast cancer cell proliferation^35,36^. There is now growing evidence that circadian disruption can interfere with cell proliferation, apoptosis, DNA damage repair, and immune functions^8,37^. It has been recognized that disrupting the expression of circadian genes, which oscillates according to the circadian rhythm, increases the risks of different types of cancer^38^. Indeed, both exposure to LAN and melatonin suppression are associated with an imbalance in the regulation of melanocyte function and the inhibition of melanin secretion in the skin with a subsequent loss of the protective effects against carcinogenic agents, such as exposure to UV radiation^39^. This type of radiation is known to have multiple effects on skin tissue, including inflammation, DNA damage and immunosuppression^40,41^. According to the Skin Cancer Foundation, approximately 86% of melanomas and 90% of non-melanoma skin cancers are associated with exposure to solar UV radiation^7^. Therefore, the increased risk of melanoma with longer duration of shift work detected in our results can be explained by intense intermittent exposure to UV radiation, which could be related to shift work. In addition, the association of lower risk of BCC with shift work demonstrated in our results can possibly be explained by shorter duration of exposure to UV radiation. Another important point that should be highlighted is the association of certain occupations, which include shift work in their schedules, such as nurses and pilots, with the risk of skin cancer. One might surmise that there are other potential factors related to these occupations that increase the risk of skin cancer, such as exposure to cosmic radiation that can cause genetic and cytogenetic damage^24,42^. Therefore, it is difficult to determine whether shift work alone or in association with other occupational factors is responsible for the increased risk of certain type of skin cancer in this population. Taken together, these factors could contribute to the progression and development of many cancers, including all types of skin cancer. Notably, the differences in the risks of BCC, SCC, and melanoma as related to shift work history suggest a variable role played by this type of work in the carcinogenesis of different types of skin cancers.

The quantified Q and I^2^ tests demonstrated statistically significant heterogeneity in the group of melanoma (I^2^ = 66.1%, p <0.001), BCC (I^2^ = 70.8%, p <0.033) and SCC (I^2^ = 76.1%, p = 0.006). In subgroup analyses of melanoma, we found that heterogeneity could be avoided among studies performed in Europe, studies that included subjects who were self-employed and workers, studies that included only males and, in the groups assessed by interview and databases. This suggests that those studies could provide more reliable evidence and that using interview and databases outperforms the use of questionnaires in the assessment of exposure. Of note, high heterogeneity in studies done in America may reflect methodological and clinical heterogeneity such as differences in the design, conduct of the studies and variations in participants’ characteristics (e.g., sex, age, baseline disease severity, ethnicity, comorbidities), interventions, exposures or outcomes evaluated^43,44^. We carried out a meta-regression analysis to assess the possible sources of heterogeneity among the variables in every study. Our results showed that only geographical region and exposure assessment might explain the heterogeneity among the enrolled studies for both melanoma and SCC. Using the leave-one-out sensitivity analysis, we found that the heterogeneity was significantly reduced if the Schernhammer 2011 study^14^ was omitted in the melanoma group, the Heckman 2017 study^15^ in the BCC group and the Lie 2007 study^21^ in the SCC group.

To the best of our knowledge, this is the first meta-analysis comprehensively focused on the associations between shift work and the risks of different types of skin cancer. There are several strengths in this meta-analysis. First, the large sample size of the studied populations collected from the included studies could help ensure more precise risk estimation and enhance the statistical power of our results. Of note, five of the included studies used databases such as the Nurses’ Health Study and Norwegian board of health’s register, which provide an excellent opportunity to study a population across these countries with high rates of follow-up. Second, eight of the included studies had a cohort design, which is well known to give more credible and valuable results when compared to case-control studies^45^. Third, eight of the enrolled studies were of high quality (NOS ≥ 7), and only one study was of low quality (NOS = 6). Finally, the included studies were conducted in different countries, which increases the generalizability of our results. Nevertheless, our study encountered many limitations. First, there was no standard definition of shift work across the enrolled studies. One of the included studies considered an employee who employed for at least one year as a flight attendant to be a shift worker^16^. Another study defined shift work as working between 1:00 AM and 2:00 AM for at least 6 months^19^. This lack of consistent definition of shift work might lead to a certain degree of misclassification and dilution of the pooled RR^26^. Second, the significantly high heterogeneity can be explained by the substantial variability in the definition of shift work, geographical region, study design, occupation, sex and exposure assessment tools. It is interesting, however, to note that these discrepancies were captured during NOS scoring as it assesses the risk of bias in each study. Therefore, our results should be interpreted with caution. Finally, four studies were included in the dose-response meta-analysis, hence limiting the reliability of the result. Because of the limited number of included studies and the high degree of heterogeneity, further well-designed large cohort studies are still warranted to confirm the findings of our analysis.

In conclusion, this meta-analysis demonstrated that shift work was potentially associated with increased risk of melanoma and decreased risks of BCC. No association between shift work and the risk of SCC was detected. Our results indicated that the risk of melanoma increases cumulatively by 2% for every year of shift work. Taken together, our findings suggest that more efforts are needed to protect the health of shift workers, such as regular follow-up in this population. Clearly, experimental studies and large cohort studies with long-term follow-up are needed to confirm our results and to elucidate the potential biological mechanisms that are related to shift work.

## Materials and Methods

### Data sources and literature search

The protocol of this systematic review was published before starting the review process on the publicly accessible website PROSPERO (http://www.crd.york.ac.uk/prospero/) with the registration number [CRD42019119583]. We planned and conducted this systematic literature search following the Preferred Reporting Items for Systematic Reviews and Meta-Analyses (PRISMA) guidelines^46^. An extensive systematic literature search updated to October 2018 was carried out. We used the following electronic databases: PubMed, Web of Science, Cochrane Library, EMBASE and Science Direct. Searches were performed using the following keywords: (“shift work” OR “night shift” OR “circadian”) AND (“Skin cancer” OR “Melanoma” OR “non-melanoma” OR “Basal cell carcinoma” OR “BCC” OR “cutaneous squamous cell carcinoma” OR “SCC”). We evaluated all possible articles by checking the titles and abstracts, and those that met the eligibility criteria were retrieved. Furthermore, we hand-searched the reference lists of the identified reviews and articles to further select any potentially relevant studies. Only articles written in English were included in our study, and no other limits were applied.

### Inclusion and exclusion criteria

The included articles were identified according to the following inclusion and exclusion criteria. The inclusion criteria were as follows: 1) studies with a cohort or case-control study design; 2) studies evaluating the possible relationships between shift work and the risks of different types of skin cancer; 3) studies with defined and quantified shift work, 4) studies with results that included the risk estimates, such as RRs, Odds rations (ORs), hazard ratios (HRs), and standardized incidence ratios (SIRs) with 95% confidence intervals (CIs) or that provided sufficient data to calculate them. The exclusion criteria were as follows: 1) studies written in language other than English; 2) studies including recurrent skin cancer; 3) studies with the same population included in more than one study; however, in the latter case, the study with the largest number of patients was selected. Investigators were divided into two groups that worked independently to evaluate articles for inclusion. In cases of doubt, full-text articles were discussed between the two groups to solve any discrepancies.

### Data extraction

The titles and abstracts of all identified articles were screened to identify all potentially eligible studies based on the predefined data extraction form. The following data were extracted: first author’s last name, year of publication, study location, study design, number of cases/number of all subjects, sex, age, occupations of participants, type of skin cancer, range of shift work, definition of shift work; tool of exposure assessment; and variables of adjustment (Table 1). Two reviewers (E.Y. and N.M.) worked independently to extract the data from all eligible studies. Group discussion with the other two investigators (N.N. and R.T.) was used to resolve any inconsistency in data extraction.

### Quality evaluation

Quality assessment of the eligible studies was conducted using the NOS^22^. It includes eight items that allows for evaluating population selection (4 items), comparability of study groups (1 item) and exposure assessment (3 items) in case-control studies. However, in cohort study, it allows for evaluating population selection (4 items), comparability of study groups (1 items) and outcome evaluation (3 items). A study can be awarded a maximum of one star for each item within the selection, exposure and outcome categories. A maximum of two stars can be given for comparability. The total number of the stars that quantitatively indicates the quality of the study is 0–9. For each paper the scores are consisting of a letter (a, b, c or d) that represents illustrative item for the NOS quality coding item list, and a number (0 or 1) indicating the score value for this illustration (Supplementary Table 1). Studies with scores ≥ 7 were designated as high quality. NOS quality evaluation was conducted independently by two investigators (E.Y., N.M.). Group discussion and consultation with the other two investigators (N.N. & R.T) were used to settle all disagreements.

### Statistical analysis

We assessed the possible associations between shift work and the risks of different types of skin cancer with STATA statistical software version 16.0 (Stata Corp, College Station, TX, USA). Studies included in this meta-analysis reported different durations of shift work (e.g. >10 years, >20 years and >2000 days) as “ever” and compared it to “never” exposure to shift work. However, only one study compared short sleep duration to 7 hours of sleep every day^25^. Because the absolute risk of skin cancer is relatively low, standardized mortality ratios, HRs, ORs and RRs were treated as equivalent measures of risk, and we used them to calculate the pooled RR^47^. A random effect model was used to combine the estimated effects. The quantified Q test and I^2^ test were used to evaluate the statistical heterogeneity among the included studies. P <0.10 and I^2^ > 50% were considered to indicate statistically significant heterogeneity^48^. A leave-one-out sensitivity analysis was performed by serially repeating the meta-analysis and leaving out exactly one study at a time. This was used to assess the potential source of heterogeneity, to evaluate the effect of one study on the overall result and to confirm that our findings were not driven by any single study. To explore the heterogeneity, we performed subgroup analyses based on geographical region, assessment of exposure, quality score, study design, sex, occupation and if the studies adjusted to exposure to UV radiation in the multivariate analysis or not. Moreover, meta-regression analysis was performed to explore heterogeneity between subgroups.

We conducted Egger’s regression test and constructed funnel plots to assess the publication bias of the enrolled studies for each subtypes of skin cancer^49^. P <0.05 suggested that there was evidence of publication bias. In addition, the trim-and-fill procedure was used to search for studies missing in the meta-analysis^50^. This test assumes a normal distribution of the effect sizes of all included studies around a central point of a graph. If asymmetry is detected, then it adjusts for the possible effect that unpublished studies could have on the result. For the dose response meta-analysis, we selected studies that included at least three categories of shift work and supplied the number of the cases in each category^26^. For this analysis, the risk estimates and the doses were re-summarized via the method proposed by Greenland and Longnecker^51^. We assigned the dose of exposure in each category as the midpoint of the lower and upper boundaries. If the upper boundary of the highest category was not provided, the scale of the interval was supposed to be the same as in the preceding category.

## Supplementary information

Supplementary information.
