# Supplementary material for: Shift work and risk of skin cancer: A systematic review and meta-analysis
Source: Sci Rep. 2020 Feb 6;10:2012. doi: 10.1038/s41598-020-59035-x (PMC7005031; doi:10.1038/s41598-020-59035-x)
Supplement: Supplementary file 1 — Supplementary information. [file 41598_2020_59035_MOESM1_ESM.pdf]

Shift work and risk of skin cancer: A systematic review and meta-analysis

Einas Yousef [1,2,*], Noha Mitwally [1], Noha Noufal [1,3], Muhammad Ramzan Tahir [4]

Quality scoring for included cohort studies using New castle-Ottawa Scale (NOS)

The NOS is a scale, which includes eight items, that allows for evaluating population selection (4 items), comparability of study groups (1 item) and exposure assessment (3 items) in case-control studies. However, in cohort study, it allows for evaluating population selection (4 items), comparability of study groups (1 items) and outcome evaluation (3 items). A study can be awarded a maximum of one star for each item within the selection, exposure and outcome categories. A maximum of two stars can be given for comparability. The total number of the stars that quantitatively indicates the quality of the study is 0-9. Studies with scores more than or equal 7 were considered of high quality. For each paper the scores are consisting of a letter (a, b, c or d) that represents illustrative item for the NOS quality coding item list, and a number (0 or 1) indicating the score value for this illustration.

No.	Reference	Selection				Comparability	Outcome			Total Score	AHRQ standard
		Representative-ness of exposed cohort	Selection of non-exposed cohort	Exposure ascertainment	Outcome of interest History	On basis of the design or analysis	Ascertainment	Follow up	Follow up adequacy		
1	Heckman 2017	c0	a1	a1 or b1*	a1	a1b1	a1	a1	a1	8	Good
2	Schernhammer 2011	c0	a1	a1	a1	a1b1	b1	a1	a1	8	Good
3	Kjaer 2007	c0	a1	a1	a1	a1b1	b1	a1	b1	8	Good
4	Yong 2014	c0	a1	a1	b0	a1b1	b1	a1	a1	7	Fair
5	Pinkerton 2018	c0	b0	a1 or b1**	b1	a1b1	b1	a1	b1	7	Fair
6	Cohen 2015	c0	c0	a1	b0	a1b1	b1	a1	a1	6	Fair
7	Schwartzbaum 2007	a1	a1	b1	b0	a1b1	b1	a1	a1	8	Good
8	Lie 2007	c0	a1	a1	b0	a1b1	b1	a1	b1	7	Fair

*Data extracted from register and questionnaire.

** Data extracted from company records and interviews.

Quality scoring for included case-control studies using Newcastle-Ottawa Scale (NOS)

No.	Reference	Selection				Comparability	Exposure			Total Score	AHRQ standard
		Case Definition	Case Representation	Control Selection	Control Definition	On basis of the design or analysis	Exposure Ascertainment	Same method of ascertainment	Response Rate		
1	Parent 2012	a1	a1	a1	a1	a1b1	a1	a1	a1	9	Good

Converting the Newcastle-Ottawa Scale (NOS) to Agency for Healthcare Research and Quality (AHRQ) standards (good, fair and poor)

Good quality: 3 or 4 stars in selection domain AND 1 or 2 stars in comparability domain AND 2 or 3 stars in outcome/exposure domain.

Fair quality: 2 stars in selection domain AND 1 or 2 stars in comparability domain AND 2 or 3 stars in outcome/exposure domain.

Poor quality: 0 or 1 star in selection domain AND 0 stars in comparability domain AND 1 or 1 star in outcome/exposure domain.
